# Image-level and group-level models for *Drosophila* gene expression pattern annotation

**DOI:** 10.1186/1471-2105-14-350

**Published:** 2013-12-03

**Authors:** Qian Sun, Sherin Muckatira, Lei Yuan, Shuiwang Ji, Stuart Newfeld, Sudhir Kumar, Jieping Ye

**Affiliations:** 1 Center for Evolutionary Medicine and Informatics, The Biodesign Institute, Arizona State University, Tempe, AZ, 85287, USA; 2 School of Life Sciences, Arizona State University, Tempe, AZ 85287, USA; 3 Ira A.Fulton Schools of Engineering, Arizona State University, Tempe, AZ, 85287, USA; 4 Department of Computer Science, Old Dominion University, Norfolk, VA, 23529, USA; 5 Center of Excellence in Genomic Medicine Research, King Abdulaziz University, Jeddah, Saudi Arabia

## Abstract

**Background:**

*Drosophila melanogaster* has been established as a model organism for investigating the developmental gene interactions. The spatio-temporal gene expression patterns of *Drosophila melanogaster* can be visualized by *in situ* hybridization and documented as digital images. Automated and efficient tools for analyzing these expression images will provide biological insights into the gene functions, interactions, and networks. To facilitate pattern recognition and comparison, many web-based resources have been created to conduct comparative analysis based on the body part keywords and the associated images. With the fast accumulation of images from high-throughput techniques, manual inspection of images will impose a serious impediment on the pace of biological discovery. It is thus imperative to design an automated system for efficient image annotation and comparison.

**Results:**

We present a computational framework to perform anatomical keywords annotation for *Drosophila* gene expression images. The spatial sparse coding approach is used to represent local patches of images in comparison with the well-known bag-of-words (BoW) method. Three pooling functions including max pooling, average pooling and Sqrt (square root of mean squared statistics) pooling are employed to transform the sparse codes to image features. Based on the constructed features, we develop both an image-level scheme and a group-level scheme to tackle the key challenges in annotating *Drosophila* gene expression pattern images automatically. To deal with the imbalanced data distribution inherent in image annotation tasks, the undersampling method is applied together with majority vote. Results on *Drosophila* embryonic expression pattern images verify the efficacy of our approach.

**Conclusion:**

In our experiment, the three pooling functions perform comparably well in feature dimension reduction. The undersampling with majority vote is shown to be effective in tackling the problem of imbalanced data. Moreover, combining sparse coding and image-level scheme leads to consistent performance improvement in keywords annotation.

## Background

The development of a multi-cellular organism begins from a single fertilized egg and proceeds as cell division and differentiation, controlled by the spatio-temporal expression of a multitude of genes over time [1]. Study of the gene regulatory network is a crucial step towards unveiling the mechanism governing cell-fate differentiation and embryonic development [2]. For many years, *Drosophila melanogaster* has been established as the canonical model organism for the study of the fundamental principles of animal development [3-5]. To facilitate a systematic understanding of transcriptional regulation during *Drosophila* embryogenesis, the Berkeley Drosophila Genome Project (BDGP) [6,7] has produced a comprehensive atlas of gene expression patterns in the form of two-dimensional (2D) digital images using high-throughput RNA *in situ* hybridization [8]. These images capture the spatial pattern of individual genes at a particular developmental stage (time). The images in BDGP are annotated with anatomical and developmental ontology terms using a controlled vocabulary to facilitate text-based search of gene expression patterns [6]. Currently, the annotation is performed manually by human curators. With the rapid accumulation of available images generated by high throughput technologies, it is imperative to design effective computational methods to automate the annotation [9,10].

In the BDGP database, a collection of images from the same developmental stage range and the same gene are annotated by multiple ontology terms. The multi-image multi-label nature of this problem poses significant challenges to traditional image annotation methodologies. This is partially due to the fact that the assignment of a particular term to a group does not imply that all images in this group are associated with this term. Hence, special formalism needs to be designed to retain the group membership information. In addition, the shape and appearance of the same body part may vary from image to image due to the effects of stochastic processes during embryogenesis and the distortions introduced by the current image acquisition techniques. Hence, invariance to local distortions is a crucial requirement for an accurate annotation system. Several prior studies on the automatic annotation of *Drosophila* gene expression images have been reported. Zhou and Peng [11] constructed their system based on the assumption that each image in the group is annotated by all the terms assigned to that group; Ji et al. [12] considered a learning framework that incorporates both the image group information and the term-term interactions; Yuan et al. [10] designed a bag-of-words based approach in which the spatial information of the images is utilized. To date, no systematic comparison between the image-level and the group-level methods has been reported. In addition, it is not clear which scheme performs the best in combining and pooling images in the same group. Moreover, little attention is paid to the highly imbalanced intrinsic structure of the gene expression pattern images, which will significantly compromise the performance of traditional learning algorithms that expect balanced class distributions or equal misclassification costs [13].

**Figure 1:**
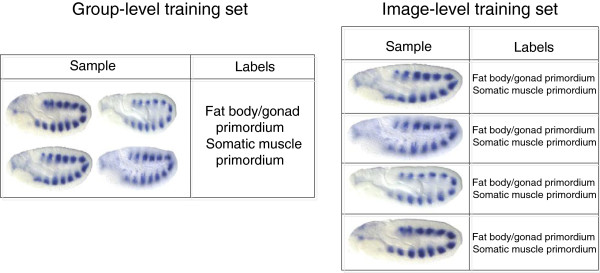
**The training process of the group-level scheme and the image-level scheme.** In the training process, one group of images is considered as one sample in the group-level scheme, while each image within a group is treated as a sample in the image-level scheme.

**Figure 2:**
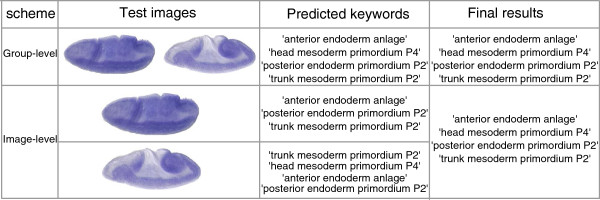
**The testing process of the group-level scheme and the image-level scheme.** In the testing stage, the group-level model will provide prediction for each group of images, while the image-level model will predict keywords for each image within this group. In addition, in the image-level scheme, we make a union of all the predicted keywords in this group, which will form the final prediction for this group of images.

In this article, we propose an image-level approach for the automated annotation of gene expression pattern images. In this approach, the images are first partitioned into several local patches; and the SIFT descriptor is used to construct a representation for each patch. We apply both bag-of-words and the sparse coding approaches to compute high-level representations from the SIFT descriptors. To obtain image features, a pooling algorithm is utilized to combine patch-level representations to generate image-level features. We propose to achieve this by a max pooling algorithm, which takes the strongest signal of the patches into consideration and is shown to perform comparably well to other pooling algorithms including average pooling and Sqrt pooling. After the features are generated, we propose an image-level scheme to perform the annotation. By assuming each image in the group contain all of the terms in the group, the training set is constructed from individual images (see Figure 1). The model built from the training set is applied to each image in the test groups. A union operation is applied to perform prediction for each group, that is, the predicted labels from all images within one group are properly combined to generate the final prediction for this group (see Figure 2). Note that our image-level representation is different from the method in [11], as we still treat a group of images as one annotation task, and our image-level annotation scheme is different from the one in [10], since we build a feature representation for each image in the group. The mechanism of undersampling is introduced in order to provide a balanced distribution for the binary classification problem. To reduce the random factor introduced by undersampling, we repeat the undersampling mechanism for multiple times and use majority vote to perform the prediction. We test our proposed approach on the BDGP images retrieved from the FlyExpress database (http://www.flyexpress.net) [14]. Annotation results from our study indicate that the image-level scheme outperforms the group-level scheme. Results also show that majority vote together with undersampling produces promising results in dealing with imbalanced data.

## Methods

In this section, we describe the image representation and keyword annotation schemes. Given a BDGP image, we apply sparse coding as well as the well-known bag-of-words (BoW) method to represent the gene expression pattern. We then employ three different pooling methods to generate the image features. Based on the image features generated, an image-level scheme and a group-level scheme are developed to annotate the keywords. The data set exhibits an imbalanced data distribution; we propose to adopt undersampling to retain the efficacy of the standard SVM classifier.

### Image representation

First, we present our framework for image representation, which is closely related to the bag-of-words (BoW) framework [15]. The BoW approach treats each image as a vector recording the frequency of every presented visual word detected from the image. The vector representation is then used to classify images into different categories [16].

**Figure 3:**
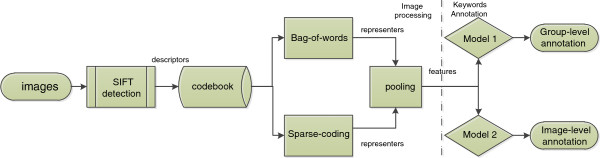
**The proposed framework of*Drosophila* gene pattern annotation.** Given an image, the SIFT detector is utilized to generate descriptors for local patches of this image. After SIFT detection, the BoW or sparse coding is applied to transform the descriptors into representers. Then the pooling functions map the representers into features. We then use these features to perform keywords annotation via different schemes.

In our framework, we first apply the SIFT descriptor to generate invariant visual features from local regions on images. A visual codebook is then constructed by applying the clustering algorithm on the invariant raw descriptors for a subset of images. The cluster centers are considered as the visual words of the images (codebook). After the codebook is generated, each descriptor will be represented by a numerical vector using either BoW or sparse coding approach. A pooling function is then adopted to summarize hundreds of representers to form one feature vector for one image. An overview of our framework is given in Figure 3.

#### Step 1: feature detection and description

The image feature detection step involves partitioning the original image into multiple regions that serve as local patches for visual description. The images in the FlyExpress database have been standardized semi-automatically, including alignment. We use a series of overlapping circles to generate multiple local patches from each image and adopt the scale-invariant feature transform (SIFT) [17] descriptor to represent each patch. SIFT converts each patch to a 128-dimensional vector. After this step, each image is represented as a collection of vectors of the same dimension (128 for SIFT). The collection of vectors are known as descriptors.

We construct the codebook based on the descriptors by selecting a subset of images and applying the k-means clustering algorithm. Visual words are then defined as the centers (or centroids) of the clusters. A visual word can be considered as a representative of several patches with the same characteristics. The codebook length is the number of clusters, which can be set manually. In our work, we set this number to 2000 as in [10]. After the codebook is constructed, the descriptor for each patch is mapped to a numerical vector based on its relationship with the codebook through two different ways: the hard assignment (BoW) and the soft assignment (sparse coding). We present both approaches in the following section.

#### Step 2: descriptor assignment

The BoW performs hard assignment for descriptors; that is, it chooses the closest visual word in the codebook to represent each descriptor. Then each image can be represented by a histogram of the visual word. Assume the number of patches for a given image is *N* and the size of the codebook is *M*. Denote *I*_*ij*_=1 if the *i*^*t* *h*^ patch is assigned to the *j*^*t* *h*^ visual word, and 0 otherwise. Then the given image can be described as *H*= [ *h*_1_,*h*_2_,…,*h*_*M*_], where

$$h_{j}=\overset{N}{\underset{i=1}{\sum }}e_{\text{ij}}.$$

A recent study [10] shows that combining BoW and spatial information would deliver better performance than using only BoW. We add spatial information into BoW in our study.

The spatial BoW can be obtained via augmenting the simple BoW representation to a larger vector with extra spatial information. We implement the spatial BoW by adopting the spatial pyramid matching scheme [18]. Denote *H* as the histogram of an image generated by a non-spatial BoW; the spatial histogram bag can be written as *S*_*n*_=[*H*_1_,*H*_2_,…,*H*_*n*_] where *n* is the number of spatial sections. In our work, we partition patches into 2 by 2 sections on each image, which enlarges the non-spatial BoW representation of the same image by a factor of 4.

The BoW approach assigns each patch to the closest visual word in the codebook, which involves solving the following optimization problem:

(1) $$\begin{array}{cc} \underset{x}{\text{minimize}}\; & \frac{1}{2}∥\text{Ax}-y{∥}_{2}^{2},\\ \text{subject to}\; & x_{i}\in \{0,1\},\overset{c}{\underset{i=1}{\sum }}x_{i}=1. \end{array}$$

It is clear that BoW is a vector quantization from *y* to the codebook, which means only one visual word can be picked to represent the patch represented by *y*. The hard assignment ignores the relationship between *y* and other visual words, while the soft assignment method overcomes the limitation with assigning each descriptor to a limited number of visual words with different weights simultaneously.

Denote the codebook matrix as $A\in R^{d\times M}$ and the descriptor for a given patch as $y\in R^{d}$, the soft assignment can be characterized as the following optimization formulation:

(2) $$\begin{array}{cc} \underset{x}{\text{minimize}}\; & \frac{1}{2}∥\text{Ax}-y{∥}_{2}^{2}+\lambda ∥x{∥}_{1},\\ \text{subject to}\; & x_{i}\geq 0,i=1,\ldots ,\mathrm{c.} \end{array}$$

where *λ* is a parameter that controls the sparsity. This is essentially a linear regression problem with *ℓ*_1_ norm regularization, known as Lasso [19] in the machine learning literature and can be solved efficiently by SLEP [20]. We also consider the spatial sparse coding, which is expected to produce a more accurate description of images.

#### Step 3: feature pooling

Next, we apply different pooling methods to transform a collection of representers to one numerical vector (the image feature). After feature detection and description, each image is represented by a collection of representers (discrete for BoW, continuous for sparse coding) of the same dimension. Pooling is used to achieve more compact representations and better robustness to noise and variance. Let *X* be the sparse coding representers of SIFT descriptors. We compute the image features by a pre-defined pooling function:

(3) z=f(X)

where *f* is applied on each column of *X*. Recall that each column corresponds to the responses of all the descriptors to one specific item in the codebook. Therefore, different pooling functions construct different image statistics. We transform a collection of representers into one vector serving as the feature of the image using three different pooling functions: average pooling, the max pooling and the square root (Sqrt) pooling.

#### Average pooling

For any image represented by a set of descriptors, we can compute a single feature vector based on some statistics of the representers. For the BoW, a common choice is to compute the histogram:

(4) $$z=\frac{1}{M}\overset{M}{\underset{i=1}{\sum }}x_{i},$$

where *x*_*i*_(*i*=1,…,*M*) is the representers generated through BoW and *M* is the number of patches we have created. In this method, we have actually taken the average value of all the BoW representers. For the more sophisticated spatial BoW, the representation *z* for one image is the concatenation of histograms associated with various locations. In this case, *z* can be seen as again a histogram after normalization. The average pooling is the most commonly used pooling functions [21-23] and it can be applied to sparse coding representers accordingly.

#### Max pooling

Average pooling can be used on both hard-assignment and soft-assignment representers. Due to the intrinsic continuity property of soft assignment, there are other ways to proceed the pooling operations. The max pooling function was introduced in [24-26], and it maps each column of *X* to its max element:

(5) $$z_{j}=\max\left\{x_{1j},x_{2j},\ldots ,x_{\text{Mj}}\right\}.$$

The max pooling basically uses the strongest signal among multiple patches to represent the information of the image, and the characteristics of that image are potentially well captured by the max pooling. Max pooling has been shown to be particularly well suited to the sparse features [27].

### Sqrt pooling

The P-norm is one of statistics that continuously transitions from average to max pooling, of which a special case is the square root of mean squared statistics (Sqrt) pooling (*P*=2). Mathematically, it is defined as:

(6) $$z_{j}=\sqrt{\frac{1}{M}\overset{M}{\underset{i=1}{\sum }}x_{\text{ij}}^{2}}.$$

It is clear that the Sqrt pooling takes advantage of all the information in *X*, and the only difference between the Sqrt pooling and average pooling lies in the statistics they choose to evaluate the information.

Each of these pooling functions captures one aspect of the statistical property of representers. We consider all three pooling functions in our empirical studies for comparison, and the results indicate that all three pooling functions perform comparably well in our application.

### Keywords annotation schemes

In this section, we describe two different annotation schemes for the keywords annotation based on various types of features extracted from the BDGP images, e.g., the max-pooling spatial sparse codes, the average-pooling BoW, etc. The group-level annotation scheme takes each group of images as one sample in the training and testing and is used in previous studies [10,12]. It has been shown to give promising results. We propose the image-level annotation scheme in this paper which treats individual image as one sample.

#### Group-level annotation

In the current BDGP database, groups of images are manually annotated with a set of keywords. It is possible that not all images in a group are associated with each keyword in the set. Following this intrinsic structure of the BDGP data, we first illustrate the group-level annotation scheme.

Given a group of images and the corresponding keywords, the SIFT descriptors are generated for each image in the group. We then perform hard assignment as well as soft assignment from the SIFT descriptors to obtain the representers. By concatenating all the representers of the group together, various pooling functions can be applied to produce the feature vector. In the group-level scheme, one group of images are treated as one sample, and the pooling functions are used to extract the information from all images within this group. We train our model using the training samples and the model is used to predict the keywords for the testing samples.

The group-level annotation scheme is built directly from the data structure, where each group of images is represented by one sample in the training and testing procedure (see Figure 1 and Figure 2). Since previous studies [10,12] have shown that the group-level scheme gives promising results in keywords annotation for *Drosophila* gene expression images, we implement the group-level scheme and use it as a baseline for comparison.

#### Image-level annotation

Different from the group-level scheme, each image serves as one sample in the image-level scheme and the pooling function is applied on an individual image rather than a group of images. In the training procedure, we assume that each image in the group is associated with all the keywords for that group. Hence, a larger number of positive training samples are generated for training our models. The training procedure is illustrated in Figure 1.

We train our model from samples representing individual images and apply it to individual images as well. After obtaining the predicted keywords for individual images, we make a union of the keywords from the same group (see Figure 2). The evaluation of the scheme is done by comparing the predicted keywords and the ground truth for that group, which is the same as group-level evaluation. The union operation is introduced to reduce the noise since not all images within a group are associated with all the keywords for that group. We also include the image-level scheme without union for comparison purpose.

Both the group-level annotation scheme and the image-level annotation scheme are implemented in our study, and our results indicate that image-level scheme outperforms the group-level scheme as it captures more information from each BDGP image.

### Undersampling and majority vote

Our image data set is highly imbalanced. For example, there are 1081 groups of images in stage range 2, while only 90 groups contain the term ‘anlage in statu nascendi’ (Table 1). It has been shown that direct application of commonly used classification algorithms on an imbalanced data set would usually provide sub-optimal results and one effective approach is to generate a balanced training set [28-30]. Intuitively, we may do random sampling to generate a balanced sample set from the imbalanced data, such as oversampling and undersampling. Oversampling adds a set *A* sampled from the minority class to the original data. In this way, the total number of samples increases by |*A*|, and the class distribution is balanced accordingly. On the contrary, undersampling removes data from the majority class and reduces the size of the original data set. In particular, denote the minority class as *B*, we randomly select a subset of size |*B*| of majority class examples while keeping the minority class untouched.

**Table 1:** Imbalanced image data set

**Stages**	**groups**	**Term1**	**Term2**	**Term3**	**Term4**	**Term5**	**Term6**	**Term7**	**Term8**	**Term9**	**Term10**
4–6	1081	302	259	231	216	199	195	107	91	90	87
7–8	877	390	371	358	342	273	241	162	145	103	71
9–10	1072	472	430	429	413	306	249	224	215	128	103
11–12	2113	936	882	604	568	554	475	284	263	261	232
13–16	2816	1068	811	791	642	564	517	492	389	353	324

In this table, we show the total number of groups and the corresponding groups which contain the top 10 terms. For most of the terms, the number of positive samples (groups which contain the term) is less than the number of negative samples (groups which do not contain the term).

In [10], oversampling is utilized to deal with imbalanced gene pattern images data set. We apply the undersampling in our study as oversampling may cause overfitting [13]. Compared with the results in [10], our experiments produce better sensitivity in prediction. To reduce the random factor in undersampling and further improve the performance, we do undersampling for multiple times and combine multiple predictions by majority vote. In our experiment, we perform undersampling for 21 times and summarize all the models to evaluate the system we have built. Majority vote [31] is adopted to perform the final prediction in our study. We use mean prediction as the baseline for evaluation and our results show that majority vote performs better.

## Results and discussion

We design a series of experiments to compare aforementioned approaches for keywords annotation, and report and analyze the experimental results. In this section, we present the comparison in four directions: 

• Comparison between spatial and non-spatial features;

•Comparison between group-level and image-level schemes;

• Comparison between BoW and sparse coding;

• Comparison of different pooling methods.

### 

#### Data description and experiment setup

The *Drosophila* gene expression images used in our work are obtained from the FlyExpress database, which contains standardized images from the Berkeley *Drosophila* Genome Project (BDGP). The *Drosophila* embryogenesis is partitioned into 6 stage ranges (1–3, 4–6, 7–8, 9–10, 11–12, 13–17) in BDGP. We focus on the later 5 stage ranges as there are very small number of keywords appeared in the first stage range.

The *Drosophila* embryos are 3*D* objects [32], and the FlyExpress database contains 2*D* images that are taken from different views (lateral, dorsal, and lateral-dorsal) [33]. As majority of images in the database are in lateral view [12], we focus on the lateral-view images in our study.

Since most keywords are stage-range specific, we build a codebook for each stage range. Based on the codebook, the spatial BoW features and the spatial sparse coding representers are computed. We adopt both group-level and image-level annotation schemes to compare the performance. Various pooling functions are applied to generate the features for annotation.

Given the feature representation, we carry out the undersampling for prediction. We focus on the most frequent 10 terms for each stage range. The groups that contain a particular term are selected as our positive samples. We use 80% of the positive samples as the positive training set *Tr*_*p*_, the remaining 20% as testing set *Tst*_*p*_. We also select a subset of samples *Tst*_*n*_(|*Tst*_*n*_|=|*Tst*_*p*_|) which do not contain the term. The test set *Tst*=*Tst*_*p*_+*Tst*_*n*_ is kept untouched during our experiment. As the data distribution is imbalanced, we apply undersamplings for 21 times on the remaining negative groups to form *Tr*_*n*_(|*Tr*_*n*_|=|*Tr*_*p*_|). Thus, we have 21 training sets *Tr*(*i*)=*Tr*_*p*_+*Tr*_*n*_(*i*), *i*=1,…,21, which are made up of the same positive set and different negative sets.

**Figure 4:**
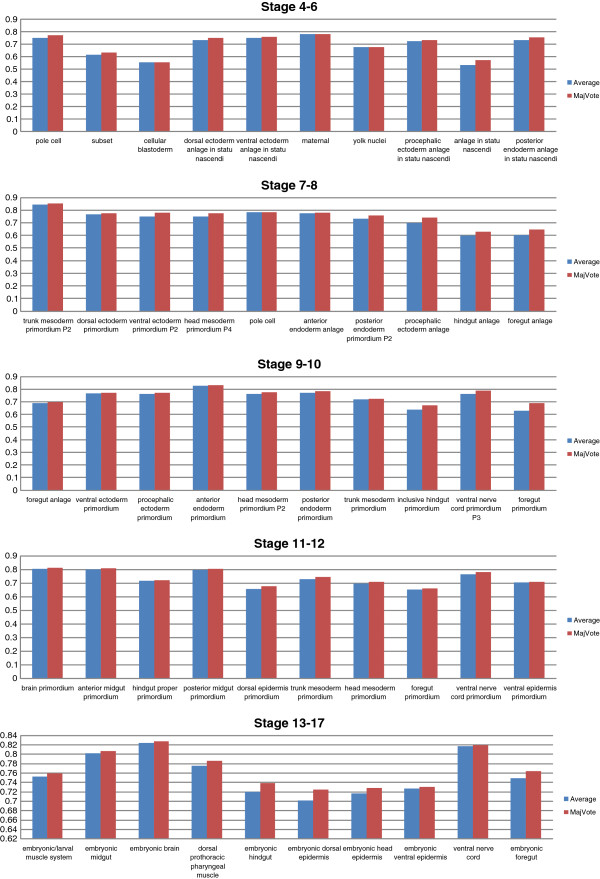
**Average vs majority vote for undersamplings.** The y-axis of the figure indicates the AUC.

We employ the one-against-rest support vector machines (SVM) [34] to annotate the gene expression pattern images, where the SVM builds a decision boundary between *Tr*_*p*_ and *Tr*_*n*_ and produces the predictions for *Tst*. The average and majority vote are used to summarize multiple undersamplings (see Figure 4). The results of the experiment verify the superiority of our proposed approach.

#### Comparison between spatial and non-spatial features

We first carry out the comparison between spatial BoW and non-spatial BoW based on the traditional group-level schemes. We fix the same settings in both cases, including the training samples, test samples and the pooling functions. The only difference between the two cases lies in the length of SIFT descriptors: the spatial descriptors is 5 times the length of those for non-spatial representation. The extra part of the descriptors captures the location information of multiple patches for the images. As the positive samples for most terms are less than the negative samples, we perform 21 under-samplings to balance the training samples for the annotation. We choose four measurements: accuracy, AUC, sensitivity and specificity to evaluate the performance of different approaches.

Comparison results for all 5 stage ranges by weighted averaging top 10 terms are shown in Table 2. From the comparison, we conclude that using spatial information improves the performance. Hence, all of the subsequent empirical studies employ the spatial representation.

**Table 2:** NonSpatial vs Spatial group-level annotation for top 10 terms in all stages

**Stages**	**NonSpatial**				**Spatial**			
	**Accuracy**	**AUC**	**Sensitivity**	**Specificity**	**Accuracy**	**AUC**	**Sensitivity**	**Specificity**
4–6	72.77	0.7573	0.7965	0.7180	73.44	0.7609	0.7965	0.7254
7–8	75.09	0.7533	0.7473	0.7626	75.74	0.7557	0.7532	0.7761
9–10	74.15	0.7499	0.7446	0.7553	74.13	0.7504	0.7429	0.7579
11–12	77.18	0.7827	0.8044	0.7610	77.74	0.7876	0.8074	0.7678
13–17	79.78	0.7988	0.8001	0.7975	80.45	0.8048	0.8042	0.8053

Spatial BOW performs slightly better than NonSpatial BOW.

#### Comparison between group-level and image-level schemes

In this experiment, we compare the group-level scheme and the image-level scheme. For the group-level scheme, a group of images serve as one sample, and the corresponding keywords act as the labels. For image-level scheme, each image of the group is treated as one sample, and the keywords of the group are assigned to all the images within the group (for training). Given a group of images, the group-level models will predict the terms associated with the whole group; the image-level models will predict the terms associated with each image within the group and the union of predicted terms from all images within the group will be used as the final prediction.

**Figure 5:**
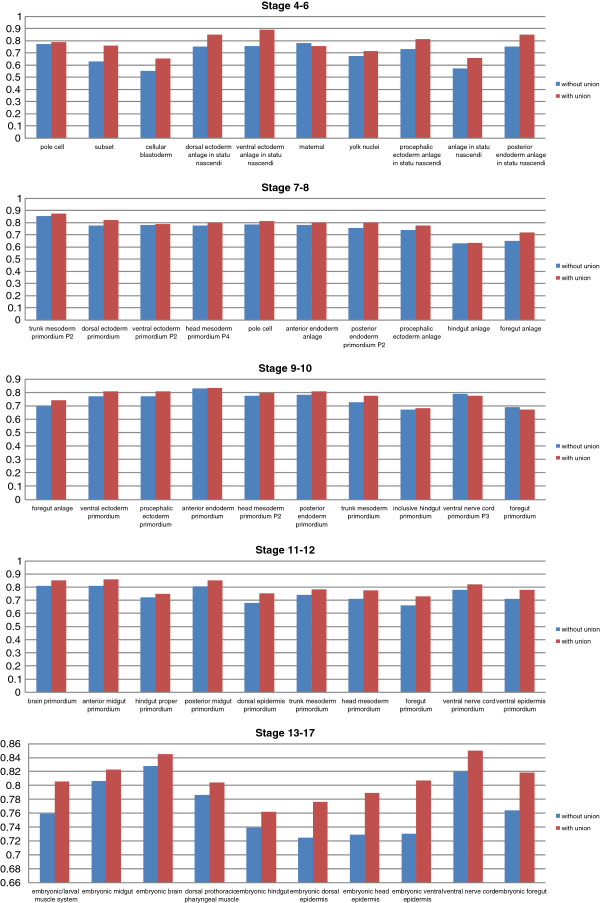
**Image-level scheme with union vs image-level scheme without union for all stage ranges.** The y-axis of the figure indicates the AUC.

To support our image-level scheme, we implement another image-level scheme without union which evaluates the prediction on the image-level. The results are reported in Figure 5. Under the same condition, the union operation significantly improves the performance of the image-level scheme over all stage ranges (see Table 3).

**Table 3:** Image-level with union vs Image-level without union

**Stages**	**Without union**				**With union**			
	**Accuracy**	**AUC**	**Sensitivity**	**Specificity**	**Accuracy**	**AUC**	**Sensitivity**	**Specificity**
4–6	69.49	0.6796	0.6623	0.6968	77.35	0.7437	0.7002	0.7872
7–8	75.78	0.7509	0.7260	0.7758	78.42	0.7666	0.7209	0.8123
9–10	74.39	0.7469	0.7373	0.7565	78.98	0.7592	0.7481	0.7702
11–12	74.69	0.7574	0.7753	0.7395	79.84	0.8021	0.8153	0.7889
13–17	78.24	0.7687	0.7433	0.7939	82.04	0.8012	0.7655	0.8359

Image-level with union performs much better than without union (sparse coding).

**Figure 6:**
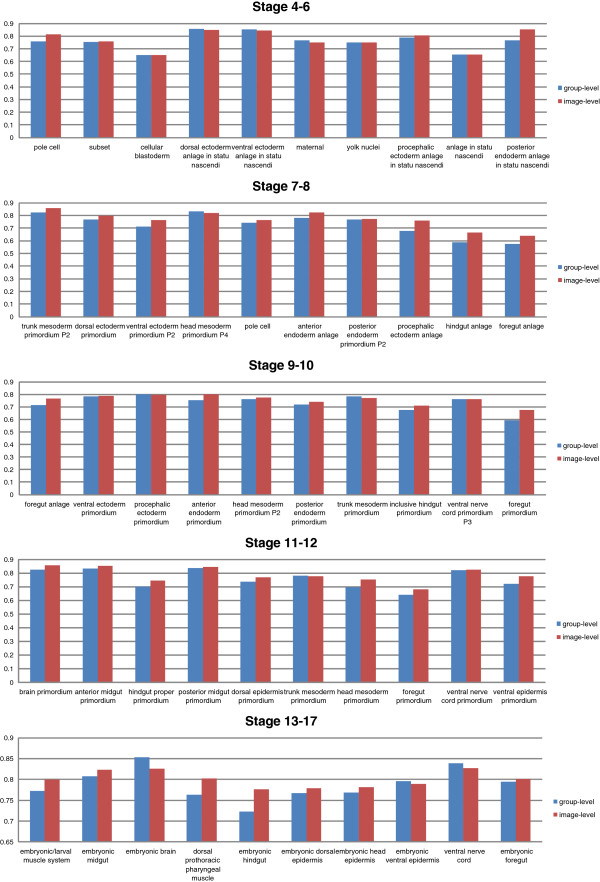
**Group-level scheme vs image-level scheme with union for all stage ranges.** The y-axis of the figure indicates the AUC.

Our empirical study also shows that the image-level scheme with union outperforms the group-level scheme, which can be seen in Figure 6. Thus, in the later part of this paper, we focus on the image-level scheme. The comparisons over all stages are summarized in Table 4.

**Table 4:** Group-level vs Image-level scheme for top 10 terms in all stages

**Stages**	**Group-level**				**Image-level**			
	**Accuracy**	**AUC**	**Sensitivity**	**Specificity**	**Accuracy**	**AUC**	**Sensitivity**	**Specificity**
4–6	73.44	0.7609	0.7965	0.7254	76.27	0.7345	0.6891	0.7798
7–8	75.74	0.7557	0.7532	0.7761	78.47	0.7635	0.7006	0.8264
9–10	74.13	0.7504	0.7429	0.7579	74.28	0.7333	0.6977	0.7669
11–12	77.74	0.7876	0.8074	0.7678	80.14	0.7974	0.7946	0.8002
13–17	80.45	0.8048	0.8042	0.8053	81.02	0.7869	0.7412	0.8325

Image-level spatial BOW performs slightly better than group-level spatial BOW.

#### Comparison between BoW and sparse coding

In this part, we compare the performances of spatial BoW and spatial sparse coding based on image-level scheme, which produces the best result in the previous study. The BoW method assigns each SIFT descriptor to its closest visual words, and uses a histogram to summarize all of the representers. On the other hand, the sparse coding assigns each SIFT descriptor to multiple visual words with different weights, and different pooling functions are used to summarize the representers. For the sparse coding, we use a subset of images to tune *λ* via cross-validation. We use average pooling for both BoW and sparse coding.

We compute the weighted average for all of the 10 terms for comparison. Overall, spatial sparse coding performs better than spatial BoW (see Table 5), which is consistent with previous studies [10].

**Table 5:** BoW vs Sparse coding for top 10 terms in all stages

**Stages**	**BoW**				**Sparse coding**			
	**Accuracy**	**AUC**	**Sensitivity**	**Specificity**	**Accuracy**	**AUC**	**Sensitivity**	**Specificity**
4–6	76.27	0.7345	0.6891	0.7798	77.35	0.7437	0.7002	0.7872
7–8	78.47	0.7635	0.7006	0.8264	78.42	0.7666	0.7209	0.8123
9–10	74.28	0.7333	0.6977	0.7669	78.98	0.7592	0.7481	0.7702
11–12	80.14	0.7974	0.7946	0.8002	79.84	0.8021	0.8153	0.7889
13–17	81.02	0.7869	0.7412	0.8325	82.04	0.8012	0.7655	0.8359

Spatial sparse coding performs slightly better than spatial BOW (image-level).

#### Comparison of different pooling methods

To study the difference of various pooling functions, we conduct another experiment to compare three different pooling methods based on spatial sparse coding. Recall that the max pooling takes the max element of each column of *X*, the average pooling takes the mean value of each column of *X*, and the Sqrt pooling take the square root of *ℓ*_2_ norm of each column of *X*. Different pooling functions utilize different statistics to summarize the representers.

Overall, the experimental results show that our image-level max-pooling approach achieves the best performance among different combinations of image representation methods and keywords annotation schemes. In particular, all three pooling methods perform comparably under the same condition. Furthermore, the max pooling and the Sqrt pooling produce slightly better results than the average pooling (see Table 6).

**Table 6:** Different pooling methods for top 10 terms in all stages

**Stages**	**Max pooling**			
	**Accuracy**	**AUC**	**Sensitivity**	**Specificity**
4–6	75.43	0.7320	0.7002	0.7637
7–8	77.28	0.7616	0.7311	0.7921
9–10	74.46	0.7457	0.7397	0.7517
11–12	78.51	0.7893	0.8015	0.7771
13–17	80.96	0.7952	0.7664	0.8240
**Stages**	**Average pooling**			
	**Accuracy**	**AUC**	**Sensitivity**	**Specificity**
4–6	75.21	0.7315	0.6976	0.7654
7–8	76.80	0.7603	0.7332	0.7874
9–10	74.32	0.7439	0.7397	0.7480
11–12	77.49	0.7828	0.8034	0.7623
13–17	80.12	0.7842	0.7521	0.8164
**Stages**	**Sqrt pooling**			
	**Accuracy**	**AUC**	**Sensitivity**	**Specificity**
4–6	75.73	0.7360	0.7031	0.7692
7–8	77.49	0.7664	0.7373	0.7955
9–10	74.58	0.7459	0.7380	0.7538
11–12	78.37	0.7907	0.8094	0.7720
13–17	80.73	0.7936	0.7681	0.8191

In our experiment, all the three pooling methods produce comparable performance. In particular, max pooling and Sqrt pooling perform slightly better than average pooling.

#### Comparison with previous work

There are three main differences between the approach in reference [10] and our paper. First, we propose different ways of pooling the representers: the sum pooling is used in [10] while we provide three different poolings in our study (average pooling, max pooling and Sqrt pooling). Secondly, we apply different ways of treating imbalanced data: the oversampling is used in [10] while we use undersampling in our paper. Finally, we adopt a different classification scheme: the group-level scheme is used in [10] while we propose the image-level scheme in our study. Compared with the results in [10], our approach produces better prediction performance (see Table 7).

**Table 7:** Comparison with previous research

**Measure**	[10]	**Our approach**
Sensitivity	0.6544	0.7946
Specificity	0.8577	0.8002

Here is a sample comparison between the previous results (refer to Table five in [10]) and our results (refer to Table 5) for stage range 11–12.

## Conclusion

This article proposes an image-level undersampling scheme for annotating *Drosophila* gene expression pattern images. In our study, images are represented by BoW and spatial sparse codes. To transform the representers to sample features, different pooling functions are adopted. In addition, an image-level annotation scheme is presented to boost the performance. The random undersampling mechanics are applied to deal with the imbalanced data in our study. Results on images from the BDGP database demonstrate the effectiveness of our proposed approach.

The current approach is only applied to the *Drosophila* embryo images in lateral view. There are also images in dorsal view and lateral-dorsal view which are not used in our study. We plan to extend the proposed method to other views of images in the future.

## Availability of supporting data

Images used in this paper are available at http://www.flyexpress.net/.

## Competing interests

The authors declare that they have no competing interests.

## Authors’ contributions

All authors analyzed the results and wrote the manuscript. JY conceived the project and designed the methodology. QS and SM implemented the programs. QS and LY drafted the manuscript. SN, SK and JY supervised the project. All authors have read and approved the final manuscript.
